# NEW CLINICAL MEASURES OF HAND AND WRIST PROPRIOCEPTION: A PILOT STUDY FOR EVALUATING DISCRIMINATIVE VALIDITY AND TEST-RETEST RELIABILITY IN INDIVIDUALS WITH WRIST DISABILITY

**DOI:** 10.2340/jrm-cc.v8.43929

**Published:** 2025-10-28

**Authors:** Maria SONTAG, Ulrik RÖIJEZON, Christina BROGÅRDH, Elisabeth EKSTRAND

**Affiliations:** 1 Department of Hand Surgery, Skåne University Hospital, Malmö, Sweden; 2 Department of Health, Education, and Technology, Luleå University of Technology, Luleå, Sweden; 3 Department of Health Sciences, Lund University, Lund, Sweden; 4 Department of Neurology, Rehabilitation Medicine, Memory Disorders and Geriatrics, Skåne University Hospital, Malmö, Sweden

**Keywords:** hand, joint, wrist, proprioception, rehabilitation, psychometrics

## Abstract

**Objective:**

To evaluate psychometric properties of newly developed hand and wrist proprioception tests.

**Design:**

Cross-sectional and test-retest comparisons.

**Subjects/patients:**

Twenty-six individuals (mean age 40 years) with wrist disability (> 3 months) due to traumatic injury or general instability.

**Methods:**

Pointing acuity (with eyes open and closed), active joint position sense (in extension, flexion, radial- and ulnar deviation) and grip force reproduction were measured by 1 rater on 2 occasions, 1 week apart. The mean absolute error was calculated for each test. Discriminative validity (affected vs non-affected hand/wrist) was evaluated by paired *t*-test and test-retest reliability with Intraclass Correlation Coefficient (ICC).

**Results:**

The pointing acuity test with eyes closed gave higher errors for the affected hand/wrist (*p* = 0.08) and good ICCs (0.80–0.85), while the test with eyes open had poor discriminative ability (*p* = 0.32) and test-retest reliability (ICC 0.13–0.16). The active joint position sense test showed higher error in flexion for the affected wrist (*p* = 0.03), and the ICC was moderate (0.51). The remaining joint directions and the grip force reproduction test had poor discriminative ability (*p* = 0.21–0.94) and poor to moderate ICCs (0.00–0.65).

**Conclusion:**

The pointing acuity test with eyes closed and the active joint position test in flexion show-ed promising results but need further evaluation in larger samples.

## LAY ABSTRACT

Wrist injuries or instability can affect proprioception – the body’s ability to sense movement, position and force – leading to poorer control of hand movements and reduced joint stability. To support recovery, proprioception training is included in rehabilitation, and accurate clinical tests are needed to measure progress. This study evaluated 3 new clinical tests of proprioception in individuals with wrist disability due to trauma or instability: pointing acuity test (with eyes open and closed) and the ability to reposition the wrist-angle and reproduce grip force (with eyes closed). The tests were analysed for their ability to detect differences between injured and uninjured wrists and their reproducibility over time. The pointing acuity test with eyes closed and the wrist-angle reposition test in flexion showed the most promising results. This suggests that selected proprioception tests may support more precise clinical assessments and enable more targeted and effective rehabilitation for individuals with wrist injuries.

Sensorimotor control involves the ability to use complex sensory information, which is crucial for controlled movements and joint stability (1). In the absence of visual input, manual tasks are largely guided by proprioception. Proprioception is sensory information, provided to the central nervous system by mechanoreceptors located in joint capsules, ligaments, skin, muscles and tendons, which enables the perception of movements, positions and forces (2).

As the wrist is a complex joint, injuries and instability may affect proprioceptive processes, which, in turn, can negatively affect movement control and joint stability (3). It has been suggested that such changes may result in a vicious circle of muscle weakness, excessive joint loading and chronic wrist pain (3, 4). In rehabilitation, the reduction of pain and the restoration of hand and wrist functioning are contingent on an improvement of proprioception (5–10). Therefore, valid and reliable outcome measures of proprioception are needed to follow treatment effects in rehabilitation.

Various aspects of proprioception can be measured in different tests, for example, in a goal-directed movement, so-called pointing acuity. This test is normally assessed in advanced laboratory equipment environments (11). However, technological progress of small optical sensors has enabled the development of new tests applicable for the clinical setting. Proprioception can also be assessed in an active joint position sense test, commonly performed manually using a goniometer, but psychometric evaluations have reported varied results ranging from poor to excellent reliability (12–15). Furthermore, proprioception can also be captured in force reproduction where the ability to reproduce submaximal grip strength is measured (16). To the best of our knowledge, only 1 study has evaluated the reproduction test psychometrically, which showed poor to moderate test-retest reliability (16). This underscores the necessity for further development, standardization and validation of proprioception tests to be used in clinical setting, as well as the evaluation of such tests for specific patient groups.

Taken together, there is a strong need for objective clinically applicable tests for examination of hand and wrist proprioception to be able to assess progress and guide treatment in rehabilitation. The tests should be able to distinguish impaired proprioceptive ability from normal function (discriminative validity) and produce similar results when performed under similar conditions (test-retest reliability). This is an important research area, as reliable and valid evaluation methods enable the development of more precisely targeted interventions for the individual and a more specific evaluation of treatment effects.

The aim of the study was to evaluate psychometric properties of newly developed hand and wrist proprioception tests in individuals with wrist disability.

## METHODS

### Study design

This pilot study includes cross-sectional and test-retest comparisons to evaluate discriminative validity and test-retest reliability for tests of proprioception in the hand and wrist.

### Participants

Participants were recruited at the Department of Hand Surgery, Skåne University Hospital in Malmö. Inclusion criteria include individuals with wrist disability (> 3 months) due to a traumatic injury or general instability diagnosed by a hand surgeon, with pain (> 2 according to Numeric Rating Scale (NRS) 0–10 (17) and self-perceived difficulties to use the hand/wrist in daily activities. Exclusion criteria include individuals with bilateral hand/wrist problems, ongoing fracture healing in the upper extremity, surgical exploration of the posterior interosseous nerve or significant other injury/disease (not related to the wrist injury) that could affect the ability to perform the tests, as well as inability to communicate in Swedish or other difficulties to understand and follow test instructions.

Before being included in the study, the participants gave informed consent to participate. The principles of the Declaration of Helsinki were followed, and this study was approved by the Swedish Ethical Review Authority 2022-09-05 (Dnr 2022-03946-01).

### Proprioception tests

**Fig. 1:**
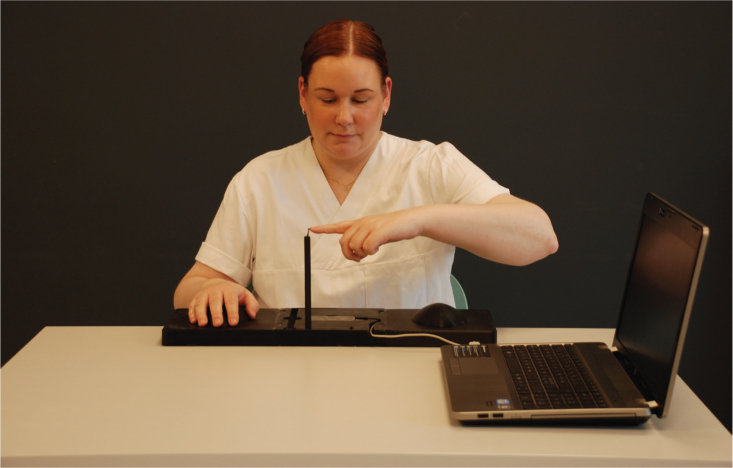
Pointing acuity test performed with an optical 3D tracking sensor (Leap Motion Controller), a computer with custom-made software and a prefabricated platform with a target (plastic stick) positioned 20 cm above the sensor.

The pointing acuity test was performed using an optical 3D tracking sensor, the Leap Motion Controller (LMC) (Ultraleap, California, United States, www.ultraleap.com) (Fig. 1), together with a custom-made software. The test was performed by moving the index finger (from a resting position on a platform) to a target (plastic stick), positioned 20 cm above the sensor, as precisely as possible (Fig. 1). To calibrate the target position of the index finger, participants were instructed to hold their finger on the target for 3 s and memorize this exact location, during which an initial measurement was taken. The test was thereafter repeated 10 times, and the pace was set by a metronome within the software, giving a beep sound at 2 s intervals. The test was first performed with eyes open and thereafter with eyes closed, with each hand. In the test with eyes closed, the target was removed to avoid any tactile input. Before the actual test, trials were performed to let the participants get familiar with the procedure. The score was calculated as the mean absolute error (AE), the absolute distance in mm between the fingertip and the target.

**Fig. 2:**
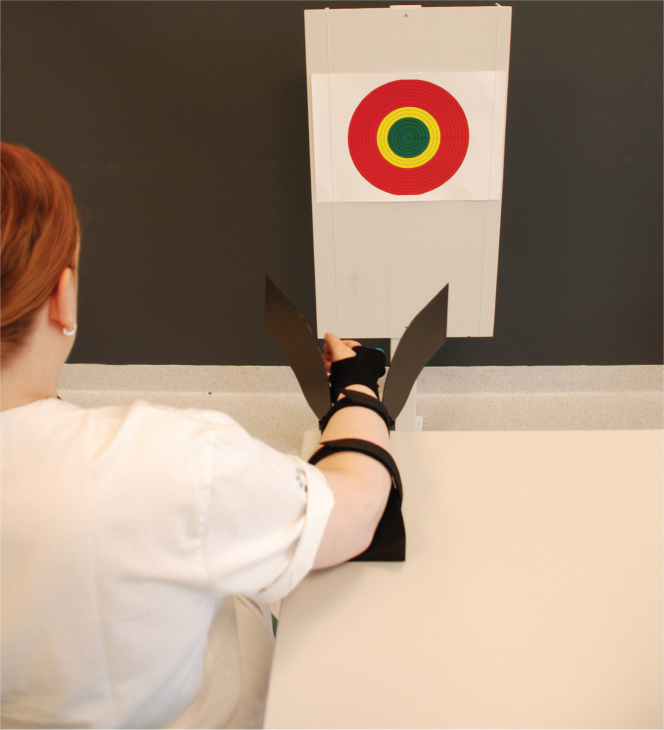
Active joint position sense test performed with a bullseye poster (268 mm diameter) positioned 90 cm in front of the participant with a laser pointer attached between metacarpal II and III.

The active joint position sense test was performed using a poster (A3 format) containing a circular diagram (268 mm diameter) with a bullseye and a laser pointer (Motion Guidance Clinical kit, Denver, United States, www.motionguidance.com) attached between the metacarpal II and III (Fig. 2). Active joint position sense was tested in 4 directions: wrist extension, flexion, radial- and ulnar deviation. The forearm was fixed on a prefabricated rig, the first carpometacarpal joint 90 cm from the diagram. First, the wrist was set in neutral position (laser beam pointing at the centre of the eye). Thereafter, the participants moved the wrist in the direction tested and returned to as close as possible to the neutral position without using vision. In extension/flexion, the movement was limited to 30 degrees by the rig, and in ulnar/radial deviation, the movement was limited by the normal range of motion. The test was repeated 6 times in each direction with reset movements (oscillations) between each repetition. Before the actual test, trials were performed to get familiar with the procedure. The score was calculated as the mean AE, the deviation from the centre of the bullseye in degrees from 0° to 9°.

**Fig. 3:**
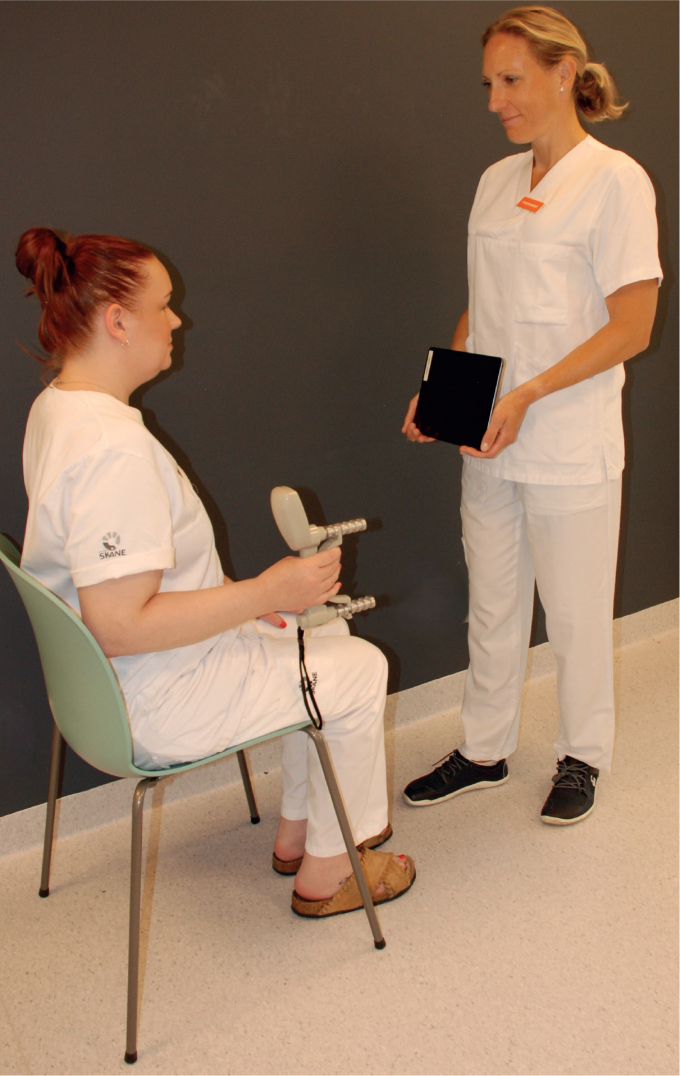
Force reproduction test performed with JAMAR Smart Hand Dynamometer together with the Jamar^®^ Smart application on a tablet.

The grip force reproduction test was performed using a digital dynamometer JAMAR Smart Hand Dynamometer (ASP Global, Austell, United States, www.aspglobal.com) together with the Jamar^®^ Smart application (ASP Global, Austell, United States, www.aspglobal.com) on a tablet (Apple, iPad, Cupertino, California, United States, www.apple.com) (Fig. 3). The standardized position (elbow in 90 degrees, and forearm and wrist in neutral position) (18) was used in the force tests. First, maximum voluntary contraction (MVC) was measured to calculate the target force, 50% of MVC. Thereafter, the participants should attain the target force with visual feedback from the tablet and memorize this force. In the reproduction test, the target force was to be reproduced without visual input. The test was repeated 3 times per hand. The score was calculated as the mean AE (kg) in relation to the target force (%).

### Procedures

First, pointing acuity was measured, followed by the tests of active joint position sense and grip force reproduction. The tests were administered by a physiotherapist with clinical expertise in hand rehabilitation (MS) at the Department of Hand Surgery on 2 different occasions (Test occasion 1, T1 and Test occasion 2, T2), approximately 1 week apart according to a standardized test protocol. The group was balanced such that every other participant started the tests with the affected hand and every other participant with the non-affected hand, on both test occasions. The test protocol took approximately 60 min to complete.

At T1, background information was collected regarding age, gender, handedness, type of wrist injury/problem, grip strength (measured by dynamometer) and wrist disability [assessed by the Patient Rated Wrist Evaluation (PRWE) (19)]. The PRWE is valid and is a reliable questionnaire consisting of 15 questions on pain (5 questions) and functioning (10 questions) (20).

### Statistical analysis

Statistical analyses were performed with the SPSS version 28.0 (IBM Corporation, Armonk, New York, United States). Probability values less than 0.05 were considered as statistically significant. For descriptive data, means (standard deviations, SD), frequencies and medians (interquartile ranges, IQR, and maximum and minimum values) were calculated. Data were judged to be normally distributed according to visual inspection of the histograms.

To evaluate discriminative validity, the measurements of the affected and non-affected hand/wrist at T1 were compared and analysed by a paired *t*-test. The test-retest reliability was evaluated by the Intraclass Correlation Coefficient (ICC) and 95% confidence interval (95% CI) and the Standard Error of Measurement (SEM). The strength of the ICC was interpreted as poor for values less than 0.5, moderate between 0.5 and 0.75, good between 0.75 and 0.9 and excellent for values greater than 0.90 (21).

## RESULTS

The demographics and clinical characteristics of the participants are presented in Table I. The mean age of the participants was 40 years (69% women) and most had a diagnosis of post-traumatic pain. MVC in grip strength was impaired in the affected hand compared to the non-affected (*p* < 0.001) and reproducible between the test occasions (ICCs 0.96–0.97).

**Table I:** Characteristics of participants (*n* = 26)

Characteristics	
Age, mean (SD; min-max)	40 (13; 20–69)
Sex (*n*)	
Men	7
Women	18
Other	1
Dominant hand, % (*n*)	
Right	100 (26)
Affected hand, % (*n*)	
Dominant	65 (17)
Non-dominant	35 (9)
Time with symptoms (months), mean (SD; min-max)	37 (56; 6–240)
Injury, % (*n*)	
Posttraumatic injury	96 (25)
General instability	4 (1)
PRWE, median (IQR)	
Total	57 (40–67)
Pain	33 (23–38)
Function	46 (35–59)
Grip strength, MVC (kg), mean (SD)	
Grip strength NA	36 (12)
Grip strength A	26 (12)

SD: standard deviation; min: minimum; max: maximum; IQR; Inter Quartile Range; MVC: Maximum Voluntary Contraction; PRWE: Patient Rated Wrist Evaluation; NA: non-affected hand/wrist; A: affected hand/wrist.

Data were collected from 26 participants at T1. At T2, 2 persons dropped out, 1 had an arm injury and 1 declined to further participate. The mean number of days between T1 and T2 was 7 days (SD 1).

For the pointing acuity test, technical problems occurred in 2 cases in both test sessions. Therefore, data from 24 participants at T1 and 22 at T2 were available. The test with eyes open showed a non-significant difference between the hands (*p* = 0.32). For eyes closed, there was a larger AE for the affected hand/wrist, albeit non-significant (*p* = 0.08) (Table II). The ICC values ranged from 0.13 to 0.16 for eyes open and 0.80 to 0.85 for eyes closed. The SEM values ranged from 9.8 to 15.3.

**Table II:** . Results of pointing acuity test

Discriminative validity (*n* = 24)	Non-affected hand/wrist Mean AE (mm) (SD)	Affected hand/wrist Mean AE (mm) (SD)	*p*-value	
Eyes open	15.7 (14.8)	12.3 (11.9)	0.32	
Eyes closed	40.9 (18.2)	52.1 (28.8)	0.08	
Test-retest (*n* = 22)	Test occasion 1Mean AE (mm) (SD)	Test occasion 2Mean AE (mm) (SD)	ICC (95% CI)	SEM
Eyes open NA	16.2 (15.7)	13.3 (12.0)	0.16 (0.00–0.66)	13.3
Eyes open A	12.9 (12.6)	11.6 (7.5)	0.13 (0.00–0.65)	9.8
Eyes closed NA	42.3 (18.8)	47.5 (23.3)	0.85 (0.64–0.94)	11.0
Eyes closed A	53.0 (28.7)	49.7 (23.8)	0.80 (0.49–0.92)	15.3

AE: absolute error; SD standard deviation; ICC: Intraclass Correlation Coefficient; SEM: standard error of measurement.

P-values for comparison between mean AE in T1 calculated by a paired-Samples *t*-test.

The active joint position sense test demonstrated a significantly larger error for the affected hand/wrist than for the non-affected in flexion (*p* = 0.03). For the other 3 directions (extension, radial- and ulnar deviation), the differences were non-significant (Table III). The ICCs of the active joint position sense tests for the non-affected hand/wrist were poor (0.00–0.49), and the SEMs ranged from 1.1 to 1.2. For the affected hand/wrist in extension, flexion and ulnar deviation, the ICCs were moderate (0.51–0.65), radial deviation showed poor ICC (0.36) and the SEMs ranged from 0.8 to 1.2.

**Table III:** Results of active joint position test

Discriminative validity (*n* = 26)	Non-affected hand/wrist Mean AE (°) (SD)	Affected hand/wrist Mean AE (°) (SD)	*p*-value	
Extension	3.5 (1.3)	3.5 (1.4)	0.94	
Flexion	3.2 (1.2)	4.0 (1.4)	0.03	
Radial deviation	3.3 (1.2)	3.2 (0.8)	0.57	
Ulnar deviation	3.6 (1.1)	3.3 (1.5)	0.21	
Test-retest (*n* = 24)	Test occasion 1Mean AE (°) (SD)	Test occasion 2Mean AE (°) (SD)	ICC (95% CI)	SEM
Extension NA	3.4 (1.2)	3.0 (1.5)	0.49 (0.00–0.78)	1.1
Extension A	3.3 (1.1)	3.2 (1.2)	0.55 (0.00–0.81)	0.9
Flexion NA	3.2 (1.2)	2.9 (1.0)	0.37 (0.00–0.73)	1.1
Flexion A	4.0 (1.5)	3.0 (1.1)	0.51 (0.00–0.78)	1.2
Radial deviation NA	3.2 (1.2)	3.0 (1.4)	0.29 (0.00–0.70)	1.2
Radial deviation A	3.2 (0.8)	2.8 (0.8)	0.36 (0.00–0.72)	0.8
Ulnar deviation NA	3.6 (1.1)	3.0 (0.9)	0.00 (0.00–0.53)	1.1
Ulnar deviation A	3.1 (1.4)	2.7 (1.0)	0.65 (0.21–0.84)	0.9

AE: absolute error; SD standard deviation; ICC: Intraclass Correlation Coefficient; SEM: standard error of measurement.

*p*-values for comparison between mean AE in T1 calculated by a paired-Samples T-test.

In the force reproduction test, the difference between the hands was non-significant, the ICCs were poor (0.36–0.45) (Table IV) and SEMs were 11.5 and 11.8, respectively.

**Table IV:** Results of grip force reproduction test

Discriminative validity (*n* = 26)	Non-affected hand/wrist Mean AE (%) (SD)	Affected hand/wrist Mean AE (%) (SD)	*p*-value	
Force sense	19.2 (13.1)	20.0 (13.4)	0.84	
Test-retest (*n* = 24)	Test occasion 1Mean AE (%) (SD)	Test occasion 2Mean AE (%) (SD)	ICC (95% CI)	SEM
Force sense NA	19.9 (13.3)	18.1 (11.9)	0.25 (0.00 – 0.68)	11.5
Force sense A	18.7 (11.7)	19.3 (13.1)	0.14 (0.00 – 0.64)	11.8

AE: absolute error; SD standard deviation; ICC: Intraclass Correlation Coefficient; SEM: standard error of measurement.

*p*-values for comparison between mean AE in T1 calculated by a paired-Samples T-test.

## DISCUSSION

This pilot study evaluated discriminative validity and intra-rater test-retest reliability for 3 newly developed tests of proprioception in the hand and wrist for the clinical setting. The pointing acuity test with eyes closed showed promising results of higher AEs for the affected hand/wrist compared to the non-affected, and the test-retest reliability was good. The results of the test with eyes open could not discriminate between hands, and the reliability was poor. For the active joint position sense test in flexion, the AE for the affected hand/wrist was significantly higher compared to the non-affected and showed moderate test-retest reliability. The other directions of the joint position sense tests had poor discriminative validity and poor to moderate reliability. The force sense test show-ed both poor discriminative ability and test-retest reliability.

Pointing acuity was measured using a small optoelectronic sensor. The test with eyes open could not identify proprioceptive impairment in the affected hand/wrist and demonstrated poor test-retest reliability. However, with eyes open, the participants could rely on vision. Conversely, with eyes closed, the accuracy of the test was dependent on proprioception, and the affected hand/wrist showed higher errors than the unaffected. The statistical difference (*p* = 0.08) in our relatively small sample size (*n* = 22) suggests that psychometric evaluation should be performed in a larger study sample. Furthermore, the reproducibility of the test with eyes closed was good. Overall, the test of pointing acuity with eyes closed appears to be a promising test of proprioception, but further evaluation is necessary before it can be implemented in the clinical setting.

Active joint position sense is commonly measured manually with a goniometer (12–15). In this study, the test was further standardized using a laser pointer and a circular diagram with a bullseye figure. The joint position test in flexion showed significantly higher errors for the affected hand/wrist, thus had the ability to discriminate between hands/wrists. This is in line with previous studies that have reported larger errors in the affected hand/wrist in active joint position sense in flexion (14, 15). The test in flexion showed moderate test-retest reliability. Previous reliability studies of active joint position tests have reported poor to moderate ICCs (0.07–0.58) (12, 13, 15), in agreement with the results of our study. Another reliability study (14) demonstrated higher ICCs (0.62–0.92); however, the retest was performed shortly after the first test (in the same session), which is questionable as recall and learning effects can improve the reliability (22). The result of the present study suggests that flexion may be the most useful direction to measure when assessing joint position sense.

The force reproduction test according to our protocol was unable to discriminate between the affected and non-affected hands, and the measurements were not reproducible. However, the maximal grip strength measurement demonstrated a capacity for distinction between the hands (lower strength for the affected hand, *p* < 0.001) and exhibited excellent test-retest reliability (ICC 0.96–0.97). The findings suggest that further refinement of the test is necessary, or that alternative methods for assessing the force sense modality should be explored.

### Clinical perspective

The wrist is a complex joint with numerous ligaments and muscles that work as static and dynamic stabilizers. As an injury to the wrist may negatively affect proprioceptive information and thereby the joint stability and sensorimotor control, it is essential to improve proprioception in wrist rehabilitation programs. Consequently, the development of valid and reliable outcome measures of proprioception is imperative to facilitate the clinical evaluation of interventions. Technological progress creates new possibilities to develop tests for the clinical setting that are more advanced.

### Strengths and limitations

A strength of the present study was that the proprioception tests were standardized concerning procedure, positioning, instructions and equipment, and that 1 examiner performed all measurements. As discriminative validity was investigated by comparing the affected and non-affected hand/wrist, cross-over effects (23) might have impaired the non-affected wrist and thereby influenced the results. Hence, future studies should evaluate discriminative validity by comparing individuals with and without injured wrists. The tests were newly developed, and therefore, their validity and reliability are not fully established. Another limitation of this pilot study was the small sample. Further development and evaluation in larger studies is needed before these new proprioception tests can be implemented and used in the clinical settings.

### Conclusion

The pointing acuity test with eyes closed and the active joint position sense test in flexion showed promising results of discriminative validity and test-retest reliability but need further evaluation in larger study samples. The other proprioception tests need additional development and evaluation before they can be used in the clinical settings.
